# *In Silico* Analysis of High-Risk Missense Variants in Human *ACE2* Gene and Susceptibility to SARS-CoV-2 Infection

**DOI:** 10.1155/2021/6685840

**Published:** 2021-04-09

**Authors:** Asmae Saih, Hana Baba, Meryem Bouqdayr, Hassan Ghazal, Salsabil Hamdi, Anass Kettani, Lahcen Wakrim

**Affiliations:** ^1^Virology Unit, Immunovirology Laboratory, Institut Pasteur du Maroc, 20360 Casablanca, Morocco; ^2^Laboratory of Biology and Health, URAC 34, Faculty of Sciences Ben M'Sik Hassan II University of Casablanca, Morocco; ^3^National Center for Scientific Technical Research (CNRST), Rabat 10102, Morocco; ^4^Department of Fundamental Sciences, School of Medicine, Mohammed VI University of Health Sciences, Casablanca, Morocco; ^5^Environmental Health Laboratory, Institut Pasteur du Maroc, 20360 Casablanca, Morocco

## Abstract

SARS-CoV-2 coronavirus uses for entry to human host cells a SARS-CoV receptor of the angiotensin-converting enzyme (*ACE2*) that catalyzes the conversion of angiotensin II into angiotensin (1-7). To understand the effect of *ACE2* missense variants on protein structure, stability, and function, various bioinformatics tools were used including SIFT, PANTHER, PROVEAN, PolyPhen2.0, I. Mutant Suite, MUpro, SWISS-MODEL, Project HOPE, ModPred, QMEAN, ConSurf, and STRING. All twelve *ACE2* nsSNPs were analyzed. Six *ACE2* high-risk pathogenic nsSNPs (D427Y, R514G, R708W, R710C, R716C, and R768W) were found to be the most damaging by at least six software tools (cumulative score between 6 and 7) and exert deleterious effect on the *ACE2* protein structure and likely function. Additionally, they revealed high conservation, less stability, and having a role in posttranslation modifications such a proteolytic cleavage or ADP-ribosylation. This *in silico* analysis provides information about functional nucleotide variants that have an impact on the *ACE2* protein structure and function and therefore susceptibility to SARS-CoV-2.

## 1. Introduction

During December 2019 in Wuhan, China, a novel infectious disease called the coronavirus disease 2019 or COVID-19 was detected and found to be caused by severe acute respiratory syndrome coronavirus 2 (SARS-CoV-2). Later on, it was declared as a pandemic by the World Health Organization (WHO) on March 2020 [1, 2]. The most common symptoms were fever, cough, shortness of breath, sputum production, and fatigue [3]. SARS-CoV-2 infects the respiratory tract and engenders acute respiratory disease [3]. SARS-CoV-2 utilizes angiotensin-converting enzyme 2 (*ACE2*) as an entry receptor and the cellular serine protease TMPRSS2 for SARS-CoV-2 spike (S) protein priming [4].

The *ACE2* gene is located on the chromosome X, specifically at the position Xp22. It contains 18 exons that show a striking resemblance in exons size and organization to those of the ACE gene [5, 6]. The complete cDNA sequence of *ACE2* encodes an 805 amino acid protein, which is composed mainly of an N-terminal signal peptide (17 amino acid residues), a peptidase domain (positioning from 19-615 amino acids), and a C-terminal collectrin, which plays a significant role as a regulator of renal amino acid transportation, insulin exocytosis, and *β*-cell proliferation [5, 7].

*ACE2* is expressed mostly in the vascular endothelial cells of the kidney and constitutes a vital site of blood pressure regulation in the renin-angiotensin system (RAS) and heart function. In the heart, *ACE2* constitutes the first pathway for the metabolism of angiotensin II and is the most essential factor for progressive cardiac disease [8–10]. Interestingly, it seems that *ACE2* has diverse biological functions, such as regulation of blood pressure by the renin-angiotensin aldosterone system (RAAS), and metabolizing angiotensin II (Ang II) to Ang (1-7), a biological active peptide that binds to the Mas receptor to exert many beneficial vasodilatory, antifibrotic, antithrombotic, and antiproliferative actions [11, 12]. Very recently, it remains possible that *ACE2* gene polymorphism may influence both the susceptibility to SARS-CoV-2 infection and COVID-19 disease outcome [5]. There are a few reports related to computational analysis of missense variants on the *ACE2* gene. Hence, the present study is aimed at identifying deleterious variants in the *ACE2* gene by using various bioinformatics tools to extrapolate the possible associations of *ACE2* polymorphisms with COVID-19 susceptibility.

## 2. Materials and Methods

### 2.1. Retrieval of Variant Datasets

The data related to *ACE2* SNPs (rsIDs) was retrieved from genome aggregation database (*gnomAD A3*) (https://gnomad.broadinstitute.org/gene/ENSG00000130234?dataset=gnomad_r3) and *Ensembl* database (https://www.ensembl.org/Homo_sapiens/Gene/Variation_Gene/Table?db=core;g=ENSG00000130234;r=X:15561033-15602148). The amino acid (AA) sequence was retrieved from UniProt (https://www.uniprot.org/uniprot/Q9BYF1.fasta). Information on human *ACE2* protein and gene was collected from the Online Mendelian Inheritance in Man (OMIM) database (https://www.omim.org/entry/300335).

### 2.2. Prediction of Deleterious nsSNPs of *ACE2* Gene

To predict the deleterious effect of nonsynonymous SNPs (nsSNPs) of the human *ACE2* gene on protein function, four tools were used, namely, SIFT, PolyPhen 2.0, PANTHER, and PROVEAN. SIFT (Sorting Intolerant from Tolerant) (https://sift.bii.a-star.edu.sg/) is a program that employs sequence homology to predict the impact of an AA substitution on protein function. It classifies AA substitutions with a score less than 0.05 as deleterious [13]. PolyPhen 2.0 (Polymorphism Phenotyping v2), (http://genetics.bwh.harvard.edu/pph2/) evaluates the impact of an AA substitution on the protein function and structure. It classifies the substitution as probably damaging (score = 1.0), and possibly damaging or benign (score = 0.0) [14]. PANTHER cSNP (Protein ANalysis THrough Evolutionary Relationship_coding_SNP) (http://pantherdb.org/tools/csnpScoreForm.jsp) calculates the likelihood of a single AA change on protein function, and it is based on the PANTHER-PSEP (Position_Specific Evolutionary Preservation) method [15]. PROVEAN (Protein Variation Effect Analyzer) (http://provean.jcvi.org/index.php) is a web server that was used to predict the impact of AA substitutions on the biological function of a protein [16].

### 2.3. Effect on the Stability of Protein

The stability of the protein was checked using the MUpro bioinformatics tool and I. Mutant Suite. MUpro (http://mupro.proteomics.ics.uci.edu/) predicts if a mutation increases or decreases the stability of protein structure [17]. I. Mutant Suite, a support vector machine-based algorithm, is available at (http://gpcr2.biocomp.unibo.it/cgi/predictors/I-Mutant3.0/I-Mutant3.0.cgi). It predicts the mutant protein stability starting from protein sequence alone [18].

### 2.4. Phylogenetic Conservational Analysis of *ACE2*

Conservation prediction of the *ACE2* protein sequence was analyzed with ConSurf (https://consurf.tau.ac.il/), a web server used for identifying functional regions in proteins by analysing the evolutionary dynamics of AA substitutions among homologous sequences. The conservation score of 1-3 is variable, 5-6 as an intermediate scale, and 7-9 as a highly conserved AA positions [19].

### 2.5. Prediction of Posttranslational Modification Sites for *ACE2*

*ACE2* protein posttranslational modification sites were predicted using ModPred (http://www.modpred.org/), a sequence-based predictor of 23 types of posttranslational modification (PTM) sites on proteins [20].

### 2.6. Statistical Analysis and Cumulative Score Calculation of Pathogenic nsSNPs

In order to predict the high-risk pathogenic nsSNPs of human *ACE2*, the cumulative score for all software tools (SIFT, PolyPhen2.0, PROVEAN, PANTHER, MUpro, I. Mutant, and ModPred) was calculated by using the Sum function in Excel [21]. Then, we set a restricted cumulative score value; when the result of the seven software tools were combined, the amino acid substitution that was evaluated to be deleterious by at least 6 tools would be identified as *ACE2* high-risk pathogenic nsSNPs. For the final, correlation analysis was performed using SPSS v19 software. ANOVA and Student *t*-tests were applied to compare the predictions of the different tools. A *p* value less than 0.05 was significant [22].

### 2.7. Modelling

The 3D structure of full-length *ACE2* protein was retrieved from The Research Collaboratory for Structural Bioinformatics Protein Data Bank (RCSB-PDB, http://rcsb.org), (ID 6M17) with a resolution of 2.9 Å. The 3D structure of the wild-type and mutant protein was generated using SWISS-MODEL (https://swissmodel.expasy.org/), and the quality of the model was checked using the Qualitative Model Energy Analysis (QMEAN) server (https://swissmodel.expasy.org/qmean/). SWISS-MODEL is a fully automated program that was used to predict the 3D structure of proteins. It generates 3D models by using homology modelling techniques [23]. The FASTA AA sequence of *ACE2* protein was an input for the SWISS-MODEL. The predicted model of *ACE2* from SWISS-MODEL was an input for the QMEAN analysis. QMEAN is a server that provides access to three scoring functions (QMEAN [24], QMEANBrane [25], and QMEANDisco) [26]. It estimates the quality of protein structure models in protein structure prediction [27]. PyMol is an open source program used for the three-dimensional visualization of macromolecules including proteins, nucleic acids, and small molecules [28]. The align command in PyMol is used to superpose two or more protein structures, and the superposition was evaluated based on RMSD (root mean square deviation) calculation [29].

### 2.8. Prediction of Structural Effect of Point Mutation on *ACE2*

Project HOPE (Have (y) Our Protein Explained) is a next-generation web server available at “https://www3.cmbi.umcn.nl/hope/” that is used to analyze the single point mutations. HOPE collects information from data sources and produces a mutation report enriched with figures that illustrates the effects of the mutation [30].

### 2.9. Prediction of Protein Interactions

*STRING* (Search Tool for Recurring Instances of Neighbouring Genes) is a web server available at “https://string-db.org/” that provides a platform for searching functional associations between proteins [31].

## 3. Results

### 3.1. SNP Dataset

In this study, 242 missense variants were collected from *Ensembl* and *gnomAD A3* databases. SIFT, PolyPhen 2.0, PROVEAN, and PANTHER algorithms were used to predict the functional effects of mutation on the protein. MUpro and I. Mutant Suite tools were used to identify the mutation effects on protein stability. HOPE, ConSurf, ModPred, SWISS-MODEL, and *STRING* were used to predict the mutation effects on protein structure, function, and protein-protein interactions. In total, 13 different bioinformatics programs and web servers were used to assess the mutation effects on the variants in this investigation, because the prediction of the effect of a mutation using one algorithm is not sufficient for assessing that mutation effect.

### 3.2. Prediction of Functional nsSNPs in *ACE2*

The twelve missense SNPs were predicted to be deleterious using SIFT (where tolerance index score was ranged from 0 to 0.02) (Table 1). The nsSNPs predicted by SIFT were validated by PolyPhen 2.0, PANTHER, and PROVEAN. In particular, PolyPhen 2.0 results showed that twelve AA switches (R219C, R219H, M383T, P389H, D427Y, R514G, R708W, R710H, R710C, R716C, L731F, and R768W) were predicted probably damaging (score > 0.96) (Table 1). The PROVEAN analysis identified seven AA substitutions (M383T, P389H, D427Y, R514G, R708W, R710C, and R768W) were scored as deleterious (score below -2.5), and the rest were noted as neutral (score above -2.5) (Table 1). PANTHER predicted that nine nsSNPs were probably damaging (R219C, R219H, M383T, P389H, R514G, R710H, R710C, L731F, and R768W), two were noted as possibly damaging (R708W, and R716C), and one was scored as probably benign (D427Y) (Table 1).

### 3.3. Effect on the Stability of Protein

MUpro and I. Mutant Suite were used to predict change in protein stability. The result of I. Mutant Suite showed that eight amino acid substitutions (R219H, D427Y, R514G, R708W, R710C, R710H, R716C, and R768W) were recorded as decreasing the stability of the *ACE2* protein, with a reliability index value ranged between 0 and 9, while the mutations (R219C, M383T, P389H, and L731F) were predicted to increase the stability of the *ACE2* protein (Table 2). The result of stability predicted by MUpro showed that all the twelve nsSNPs (R219C, R219H, M383T, P389H, D427Y, R514G, R708W, R710H, R710C, R716C, L731F, and R768W) have decreased the stability of the *ACE2* protein (Table 3).

### 3.4. Phylogenetic Conservation

**Figure 1:**
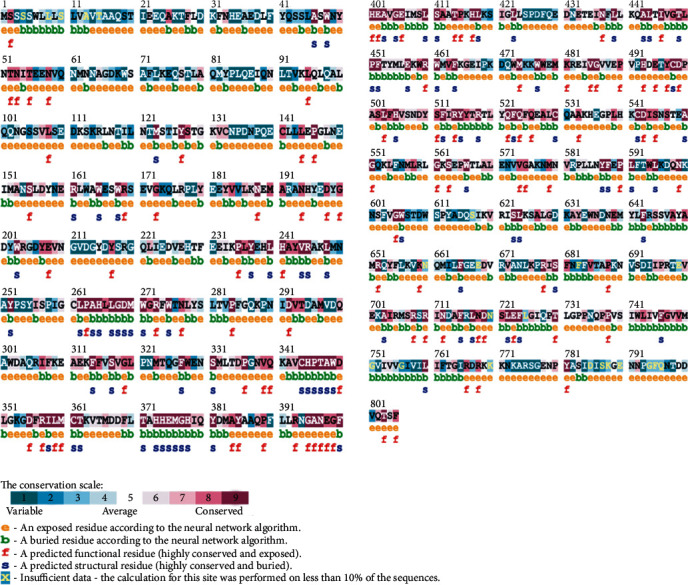
ConSurf analysis of *ACE2*_HUMAN angiotensin-converting enzyme 2 (UniProt ID: Q9BYF1).

According to ConSurf analysis, R514, R708, R710, and R768 are highly conserved residues with conservation score equal to 9 and also predicted to be exposed and functional. M389 and L731 are slightly conserved residues and buried. R219, P389, and D427 are variable residues and exposed, and finally one residue (R716) is average (conservation score equal to 5). Results of ConSurf prediction of *ACE2* SNPs are summarized in Figure 1.

### 3.5. Prediction of Posttranslational Modification (PTM) Sites

ModPred was used to predict the effect of nsSNPs on posttranslational modification (PTM) process of the human *ACE2* protein. ModPred identified sites for methylation (R219), ADP-ribosylation (R219, R708, and R768), and Proteolytic cleavage (P389, D427, R708, R710, R716, and R768). The results of PTM sites prediction are shown in Table 4.

### 3.6. Statistical Analysis and Cumulative Score Calculation of Pathogenic nsSNPs

As a result of combining seven algorithms, the amino acid substitution, namely, R708W, was scored as the most deleterious nonsynonymous SNP with a cumulative score of 7 by all the seven tools, while 6 (R219H, D427Y, R514G, R710C, R716C, and R768W), 4 (R219C, M383T, P389H, and R710H), and 1 (L731F) variant got a cumulative score of 6, 5, and 4, respectively (Table 5). Among these variants, six nsSNPs (D427Y, R514G, R708W, R710C, and R768W) are newly evaluated as *ACE2* high-risk pathogenic nsSNPs and were selected for further investigation. The prediction from SIFT, PROVEAN, PolyPhen 2.0, PANTHER, ModPred, I. Mutant, and MUpro were shown to be significant with a *p* value equal to 6.1308*E*-29 of ANOVA test and correlated (Supplementary figure 1). Student *t*-test results between the software tools were significant with a *p* value less than 0.0001, suggesting that the selected algorithms are accurate enough to evaluate the pathogenicity of these nsSNPs.

### 3.7. 3D Modelling and Biophysical Validation of *ACE2*

**Figure 2:**
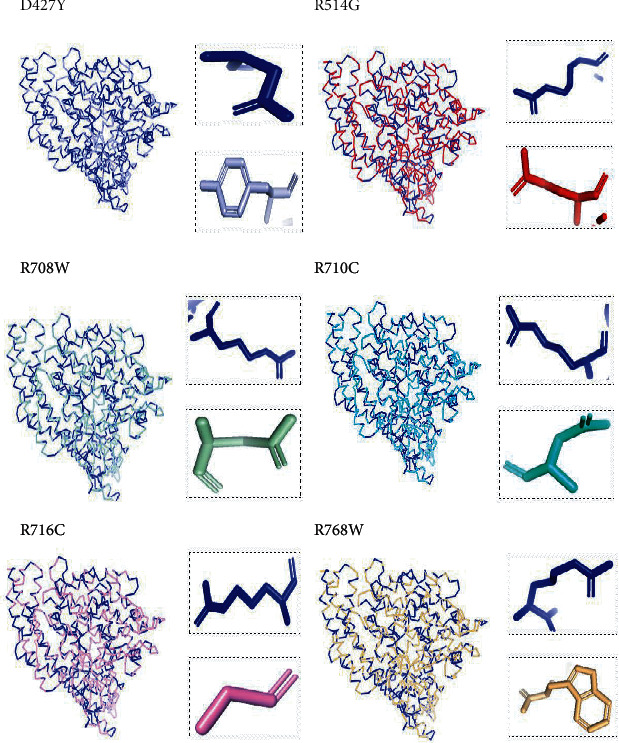
Superimposed structure of native and mutant models of the *ACE2* protein. The superimposed structure of native amino acid aspartic acid (blue color) with mutant amino acid tyrosine (light blue color) at position 427. The superimposed structure of native amino acid arginine (blue color) with mutant amino acid glycine (red color) at position 514. The superimposed structure of native amino acid arginine (blue color) with mutant amino acid tryptophan (pale green color) at position 708. The superimposed structure of native amino acid arginine (blue color) with mutant amino acid cysteine (green cyan color) at position 710. The superimposed structure of native amino acid arginine (blue color) with mutant amino acid cysteine (violet color) at position 716. The superimposed structure of native amino acid arginine (blue color) with mutant amino acid tryptophan (light orange color) at position 768.

The crystal structure of the *ACE2* protein was retrieved from the Protein Data Bank (PDB ID: 6 M17, resolution at 2.9 Å) in complex with SARS-CoV-2 receptor-binding domain and sodium-dependent neutral amino acid transporter B(0)AT1. The structure was used as a template for the comparative modelling of mutant structures through SWISS-MODEL by submitting the FASTA AA sequence of *ACE2* (percent identity equal to 100). The QMEAN server was used to predict the quality of the models, where the global QMEAN scores were 0.83 ± 0.05. This indicated that the predicted models were of good quality (Table 6). The six tested mutants, namely, D427Y, R514G, R708W, R710C, R716C, and R768W, were subjected to PyMol using “*Align command*” and compared to the native structure with regard to conformational variations by calculating the RMSD (root mean square deviation) (Table 7 and Figure 2). Project HOPE was used to identify the structural effects of mutations of interest. Results of project-HOPE of *ACE2* SNPs were displayed in Table 8.

### 3.8. Analysis of Protein-Protein Interaction

**Figure 3:**
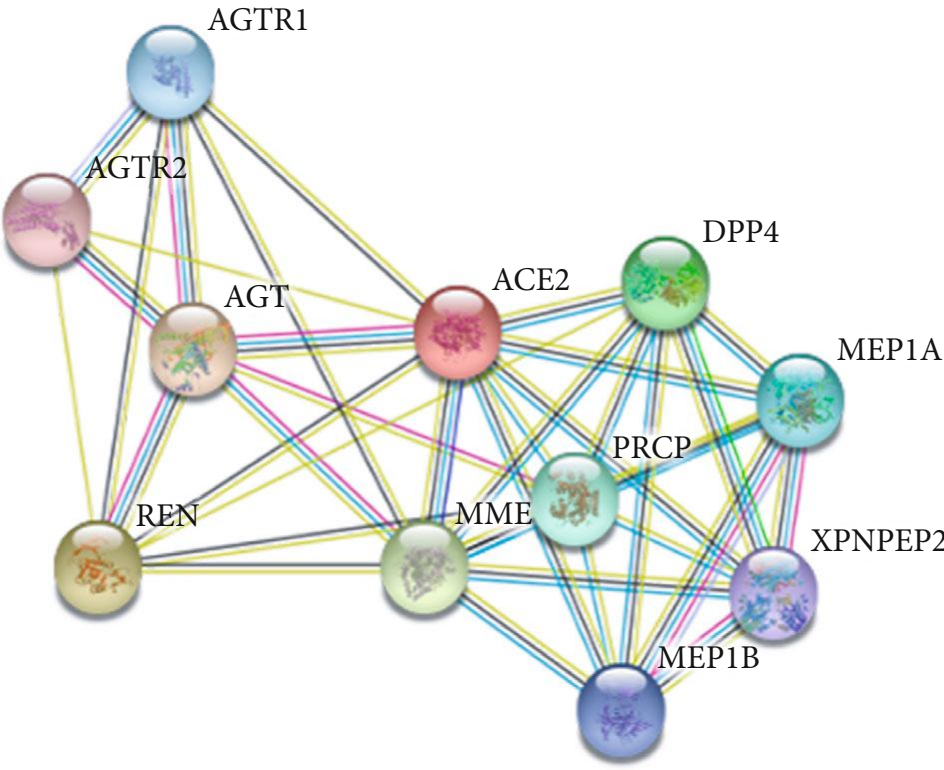
*STRING* network analysis of the *ACE2* gene.

*STRING* prediction showed that *ACE2* interacts with angiotensin II receptor type 1 (AGTR1), angiotensin II receptor type 2 (AGTR2), prolyl carboxypeptidase (PRCP), renin (REN), angiotensinogen (AGT), membrane metalloendopeptidae (MME), dipeptidyl peptidase 4 (DPP4), meprin A subunit alpha (MEP1A), meprin A subunit beta (MEP1B), and X-prolyl aminopeptidase 2 (XPNPEP2) (Figure 3).

## 4. Discussion

Angiotensin-converting enzyme 2 (*ACE2*) plays an important role in the renin-angiotensin aldosterone system by metabolizing angiotensin II to angiotensin (1-7) [11]. This important enzyme was identified as a functional receptor for the severe acute respiratory syndrome coronavirus (SARS-CoV) and the novel coronavirus (SARS-CoV-2) [11]. Some studies suggest that the receptor-binding domains of SARS-CoV-2-S and SARS-CoV-S bind with identical affinities to *ACE2* [25]. It has been shown that *ACE2* genomic variants may play a key role in susceptibilities to COVID-19 [33].

The SARS-CoV-2 starts its infection by binding to *ACE2* via its receptor-binding domain (RDB) [34]. Recently, it has been reported that the SARS-CoV-2 uses two mechanisms of host cell entry: the first mechanism on the *ACE2* mediated virus endocytosis by using the clathrin- and caveolae-dependent pathways. The second one is dependent on the transmembrane serine protease 2- (TMPRSS2-) mediated membrane fusion [34].

The study by Shang et al. 2020 [35], Walls et al. [36], Wrapp et al. 2020, and Yan et al. 2020 have demonstrated that there are important *ACE2* residues, namely, S19, Q24, T27, F28, D30, K31, H34, E35, E37, R357, E329, N330, K353, G354, D355, R357, P389, and R393, that play crucial roles in mediating the SARS-CoV-2 spike protein-*ACE2* interaction. Other results were broadly in line with *ACE2* variants that are identified to alter the virus host interaction and consequently alter susceptibility to SARS-CoV-2 [37]. Some of them, like S19P, I21V, E23K, K26R, T27A, N64K, T92I, Q102P, and H378R, are predicted to increase susceptibility and therefore render individuals more susceptible to the SARS-CoV-2 [37]. Other *ACE2* variants such K31R, N35I, H34R, E35K, E37K, D38V, Y50F, N51S, M62V, K68E, F72V, Y83H, G326H, G352V, D355N, Q388L, and D509Y are predicted to decrease susceptibility and render individuals more resistant to the SARS-CoV-2 [37]. A number of 61 deleterious variants in the *ACE2* gene including R219C, R219H, M383T, P389H, D427Y, R514G, R708W, R710H, R710C, R716C, L731F, and R768W, have been reported to influence susceptibility to COVID-19 (Hou et al. 2020). To confirm these results, the twelve variants, namely, R219C, R219H, M383T, P389H, D427Y, R514G, R708W, R710H, R710C, R716C, L731F, and R768W, were tested. This *in silico* analysis might be helpful in understanding the effect of missense variants on protein structure, function, and stability of *ACE2* in relation with COVID-19.

A 2020 study by Fahd Al-Mulla et al. has shown that the arginine residues at 708 and 716 positions play an important role in *ACE2* cleavage by TMPRSS2 and TMPRSS11D, whereas mutations on R708 and R716 seem to reduce directly *ACE2* cleavage by TMPRSS2 [32]. Another line of research (Hossein Lanjanian et al. 2021) suggests that the arginine residue at 710 position of the ACE2 receptor plays a crucial role in mediating ACE2-TMPRSS2 interaction by involving hydrogens bonds [38]. A second study achieved by Behrooz Darbani (2020) has identified the rs1316056737 (R514G) as an interaction inhibitor variant that might impact the interaction between the *ACE2* receptor and the viral spike protein [39].

In the current study, various algorithms, namely, SIFT, PolyPhen 2.0, PROVEAN, PANTHER, I. Mutant Suite, MUpro, and ConSurf, were used to identify the most deleterious nsSNPs of the *ACE2* gene. In our *in silico* analysis, we identified six nsSNPs (D427Y, R514G, R708W, R710C, R716C, and R768W) from twelve nsSNPs. The six nsSNPs were predicted to be deleterious by at least six algorithms, decreased the stability of *ACE2* by both MUpro and I. Mutant, and located in conserved regions with a score conservation of 9, expected D427Y, which may affect the structure and function of *ACE2* protein.

ModPred was used to determine the posttranslational modification (PTM) sites of *ACE2* identified R710 and R716 as PTM sites for proteolytic cleavage and R708 for ADP-ribosylation and proteolytic cleavage. Consequently, mutations at R708, R710, and R716 might affect PTM of the *ACE2* gene.

The structural deviations between the native and modelled mutants were analyzed using the RMSD by measuring the average distance between the atoms of the superimposed proteins [40]. It has also been shown that RMSD values greater than 0.15 were evaluated to be significant and would have an impact on protein function and structure [40]. Therefore, the six models R427Y, R514, R708W, R710C, R716C, and R768W showed lower RMSD values, i.e., 0.010, 0.112, 0.112, 0.112, 0.112, and 0.010, respectively, which indicate minimal structural dissimilarity between the native and mutant models of *ACE2*.

According to HOPE, the AA substitution (R514G) introduces a glycine (flexible) and could disturb the required rigidity of the *ACE2* protein and could affect the binding site where the mutation being located, while the mutation R716C is located in a region essential for cleavage by TMPRSS11D and TMPRSS2 proteases, and the difference in AA properties can disturb this region and its function.

Furthermore, by using the *STRING* server, *ACE2* protein interacts with ten various proteins, namely, AGTR1, AGTR2, PRCP, REN, AGT, MME, DPP4, MEP1A, MEP1B, and XPNPEP2. The majority of those proteins play a crucial role in the regulation of blood pressure and kidney pathways, suggesting the association of *ACE2* in kidney diseases. All the ten various proteins had a score ranging from 0.991 to 0.858, suggesting that proteins are partially biologically connected with an PPI enrichment *p* value equal to 1.29*E*-09. The proteins AGT, REN, AGTR1, and AGTR2 were found to have an important role in RAS signalling pathways such as regulation of blood pressure, body fluid and electrolyte homeostasis, and sodium retention by the kidney. The interaction analysis using the *STRING* server suggested that the *ACE2* protein not only participate in hypertension and cardiovascular diseases but also a candidate in COVID-19 infection.

However, these findings showed that D427Y, R514G, R708W, R710C, R716C, and R768W, are the structurally and functionally most significant variants in the human *ACE2* gene. (Table 7). It has been found that the residues ARG 708, 710, and 716, are located in the dimeric interface of human *ACE2*, and they are found to be important for its cleavage by the protease TMPRSS2, and this processing is indispensable for augmentation of SARS-S-driven entry into host cells [9]. The limitation of this *in silico* analysis is that the six deleterious variants should be confirmed with future extensive experiments and clinical wet lab approaches to figure out the mechanism of these mutations in susceptibility to COVID-19.

## 5. Conclusion

Our present *in silico* analysis of nsSNPs of the human *ACE2* gene concluded that the mutations D427Y, R514G, R708W, R710C, R710H, and R716C are the most deleterious nsSNPs among the reported *ACE2* gene variants. All six nsSNPs were predicted to be damaging and also affecting conserved positions, protein stability, and the posttranslational modification sites. Therefore, these mutations would likely affect the function on *ACE2* with regard to host susceptibility to SARS-CoV-2 infection. Hence, the results of this study confirm previous findings and may be helpful for further understanding the role of these six *ACE2* nsSNPs in susceptibility to COVID-19.

## Figures and Tables

**Table 1:** Summary of prediction results for nsSNPs in the human *ACE2* gene using various bioinformatics tools.

SIFT				PolyPhen 2.0		PROVEAN		PANTHER	
SNPs ID	AA variant	Score	Prediction	Score	Prediction	Score	Prediction	PSPE	Prediction
rs372272603	R219C	0	Del	1.000	Pro Dam	-2.483	Not Del	750	Pro Dam
rs759590772	R219H	0	Del	0.988	Pro Dam	-1.520	Not Del	750	Pro Dam
rs1396769231	M383T	0	Del	1.000	Pro Dam	-4.797	Del	911	Pro Dam
rs762890235	P389H	0	Del	0.993	Pro Dam	-7.862	Del	1037	Pro Dam
rs1316056737	D427Y	0	Del	0.970	Pro Dam	-3.390	Del	176	Not Del
rs1352194082	R514G	0.02	Del	0.989	Pro Dam	-6.483	Del	1037	Pro Dam
rs776995986	R708W	0.01	Del	1.000	Pro Dam	-3.105	Del	361	Pos Del
rs901495523	R710C	0	Del	1.000	Pro Dam	-2.936	Del	750	Pro Dam
rs370187012	R710H	0	Del	1.000	Pro Dam	-1.788	Not Del	750	Pro Dam
rs144869363	R716C	0.01	Del	0.975	Pro Dam	-1.638	Not Del	220	Pos Del
rs759590772	L731F	0	Del	0.995	Pro Dam	-1.124	Not Del	750	Pro Dam
rs372272603	R768W	0	Del	1.000	Pro Dam	-2.822	Del	750	Pro Dam

AA variant: amino acid variant; Del: deleterious; Not Del: not deleterious; Pro Dam: probably damaging; Pos Dam: possibly damaging; PSPE: position-specific evolutionary preservation.

**Table 2:** Prediction of effect of nsSNPs on protein stability using I. Mutant Suite.

SNPs ID	Amino acid substitution	Stability	RI	DDG value prediction (kcal/mol)
rs372272603	R219C	Increase	0	-0.50
rs759590772	R219H	Decrease	3	-0.58
rs1396769231	M383T	Increase	2	-0.32
rs762890235	P389H	Increase	2	0.20
rs1316056737	D427Y	Decrease	3	-0.56
rs1352194082	R514G	Decrease	8	-1.84
rs776995986	R708W	Decrease	4	-0.68
rs901495523	R710C	Decrease	8	-1.72
rs370187012	R710H	Decrease	9	-1.91
rs144869363	R716C	Decrease	7	-2.20
rs759590772	L731F	Increase	4	0.01
rs372272603	R768W	Decrease	0	-0.55

RI: reliability index; DDG: the free energy change value.

**Table 3:** Prediction of effect of nsSNPs on *ACE2* protein stability using MUpro.

			Method 1: SVM (support vector machine)		Method 2: neural network	
Mutation	Delta G	Prediction	Confidence score	Effect	Confidence score	Effect
R219C	-0.89	Decrease	-0.082	Decrease	-0.57	Decrease
R219H	-1.16	Decrease	-0.089	Decrease	-0.51	Decrease
M383T	-1.56	Decrease	-0.94	Decrease	-0.70	Decrease
P389H	-0.45	Decrease	-0.55	Decrease	-0.85	Decrease
D427Y	-0.79	Decrease	0.16	Increase	-0.67	Decrease
R514G	-1.15	Decrease	-0.76	Decrease	-0.98	Decrease
R708W	-0.99	Decrease	-0.88	Decrease	-0.98	Decrease
R710C	-1.17	Decrease	-0.83	Decrease	-0.87	Decrease
R710H	-1.66	Decrease	-0.07	Decrease	-0.98	Decrease
R716C	-1.34	Decrease	-0.10	Decrease	-0.82	Decrease
L731F	-0.87	Decrease	-0.04	Decrease	-0.66	Decrease
R768W	-1.89	Decrease	-1	Decrease	-0.99	Decrease

Delta G: energy change value; Confidence score: (>0: increase the stability, <0: decrease the stability).

**Table 4:** ModPred analysis for posttranslational modification sites (PTMs) prediction.

Residue	Modification	Score	Confidence
R219	Proteolytic cleavage	0.70	Medium
	ADP-ribosylation	0.67	Medium
	Methylation	0.76	Medium
P389	Proteolytic cleavage	0.52	Low
D427	Proteolytic cleavage	0.73	Medium
R708	Proteolytic cleavage	0.76	Medium
	ADP-ribosylation	0.67	Medium
R710	Proteolytic cleavage	0.54	Low
R716	Proteolytic cleavage	0.77	Medium
R768	Proteolytic cleavage	0.84	Medium
	ADP-ribosylation	0.66	Low

**Table 5:** Cumulative score calculation of tested nsSNPs.

Mutation	SIFT	PolyPhen	PROVEAN	PANTHER	I.Mutant	MUpro	ModPred	Cumulative score
R219C	1	1	0	1	0	1	1	5
R219H	1	1	0	1	1	1	1	6
M383T	1	1	1	1	0	1	0	5
P389H	1	1	1	1	0	1	0	5
D427Y^∗∗^	1	1	1	0	1	1	1	6
R514G^∗∗^	1	1	1	1	1	1	0	6
R708W^∗^	1	1	1	1	1	1	1	7
R710C^∗∗^	1	1	1	1	1	1	0	6
R710H	1	1	0	1	1	1	0	5
R716C^∗∗^	1	1	0	1	1	1	1	6
L731F	1	1	0	1	0	1	0	4
R768W^∗∗^	1	1	1	1	1	1	0	6

^∗^The highly pathogenic nsSNPs by all the seven tools, ^∗∗^the highly pathogenic nsSNPs by at least 6 tools, 0 indicates neutral prediction, 1 indicates deleterious prediction.

**Table 6:** Data generated from SWISS-MODEL.

SNPs	QMEAN	CBeta interaction energy	All-atom pairwise energy	Solvation energy	Torsion energy
D427Y	-1.09	1.08	1.05	0.76	-1.52
R514G	-1.27	0.39	0.78	0.52	-1.50
R708W	-1.28	0.33	0.70	0.52	-1.50
R710C	-1.37	0.34	0.74	0.42	-1.55
R716C	-1.33	0.42	0.76	0.45	-1.54
R768W	-1.09	1.01	1.05	0.74	-1.50

CBeta interaction energy: distance-dependent potential using CBeta atoms as interaction center; All-atom pairwise energy: assessment of long-range interactions; Solvation energy: description of the burial status of the residues; Torsion energy: analysis of the local backbone geometry [32].

**Table 7:** Structural alignment comparing mutant and wild-type *ACE2* models.

Position	Variant	Align	RMSD (Å)
427	D427Y	6 m17.1. *E*	0.010
514	R514G	6 m17.1. *E*	0.112
708	R708W	6 m17.1. *E*	0.112
710	R710C	6 m17.1. *E*	0.112
716	R716C	6 m17.1. *E*	0.112
768	R768W	6 m17.1. *E*	0.010

**Table 8:** *ACE2* protein phenotype feature prediction by HOPE analysis.

Residue	Structure	Properties
D427Y	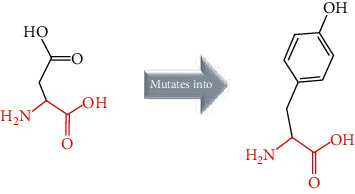	(i) The WT is predicted to be located in its preferred secondary structure, a turn the mutant prefers to be in another secondary structure, therefore the local conformation will be slightly destabilized (ii) Mutation of the WT into none has the following effect SPD-PSN: slightly inhibits interaction with SARS-CoV spike gp (iii) The mutation is possibly damaging to the protein
R514G	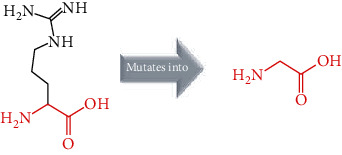	(i) The mutation introduces a glycine at this position. Glycine is very flexible and can disturb the required rigidity of the protein (ii) Residues in the vicinity of the mutated residue are annotated in the UniProt as being a binding site (iii) The mutation could affect the local structure and as a consequence affect the binding site (iv) The mutation is possibly not damaging to the protein (v) The mutant is smaller than the WT; this might lead to loss of interaction
R708W	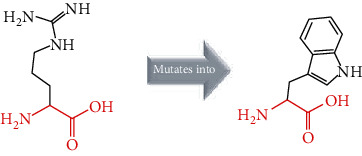	(i) The mutant is more hydrophobic than the WT (ii) The WT charge was positive, and the mutant charge is neutral (iii) The mutant is bigger; this might lead to bumps
R710C	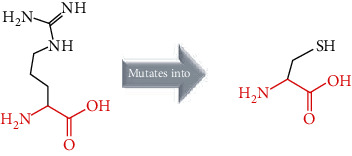	(i) The mutant is more hydrophobic than the WT (ii) The WT is very conserved (iii) The mutant is smaller; this might lead to loss of interaction
R716C	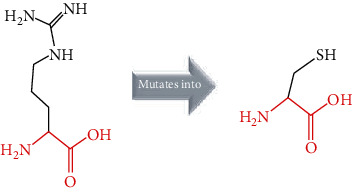	(i) The mutant is smaller than the WT (ii) The mutation is located within a stretch of residues annotated in UniProt as a special region: essential of cleavage by TMPRSS11D and TMPRSS2 (iii) The difference in amino acid properties can disturb this region and its function
R768W	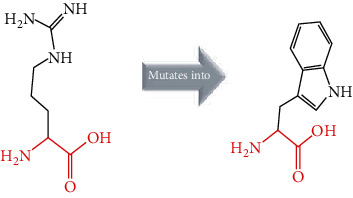	(i) The WT is very conserved. In some rare cases, the mutation might occur without damaging the protein (ii) The mutant is bigger than the WT; this might lead to bumps (iii) The mutation introduces a more hydrophobic residue at this position; this can result in loss of hydrogen bonds and or disturb correct folding

## Data Availability

All data used in this study are included within the article and supplementary information file.

## Supplementary Materials

Supplementary Materials Supplementary Figure 1: correlation analysis among predictions of pathogenic nsSNPs in the *ACE2* gene by using various software tools.
