# Supplementary material for: In Silico Analysis of High-Risk Missense Variants in Human ACE2 Gene and Susceptibility to SARS-CoV-2 Infection
Source: Biomed Res Int. 2021 Apr 9;2021:6685840. doi: 10.1155/2021/6685840 (PMC8040925; doi:10.1155/2021/6685840)
Supplement: Supplementary Materials — Supplementary Figure 1: correlation analysis among predictions of pathogenic nsSNPs in the ACE2 gene by using various software tools. [file 6685840.f1.docx]

| **Correlation** | | | | | | | | |
| --- | --- | --- | --- | --- | --- | --- | --- | --- |
|  | | SIFT | PolyPhen | PROVEAN | PANTHER | I.Mutant | MUpro | ModPred |
| SIFT | Pearson correlation | 1 | -,228 | -,296 | -,045 | -,520 | ,003 | -,205 |
|  | Sig. (bilateral) |  | ,476 | ,350 | ,890 | ,083 | ,992 | ,522 |
|  | N | 12 | 12 | 12 | 12 | 12 | 12 | 12 |
| PolyPhen | Pearson correlation | -,228 | 1 | -,029 | ,598^*^ | ,191 | -,284 | -,274 |
|  | Sig. (bilateral) | ,476 |  | ,930 | ,040 | ,552 | ,372 | ,389 |
|  | N | 12 | 12 | 12 | 12 | 12 | 12 | 12 |
| PROVEAN | Pearson correlation | -,296 | -,029 | 1 | -,519 | -,207 | -,350 | ,337 |
|  | Sig. (bilateral) | ,350 | ,930 |  | ,084 | ,518 | ,265 | ,284 |
|  | N | 12 | 12 | 12 | 12 | 12 | 12 | 12 |
| PANTHER | Pearson correlation | -,045 | ,598^*^ | -,519 | 1 | ,218 | -,032 | -,613^*^ |
|  | Sig. (bilateral) | ,890 | ,040 | ,084 |  | ,495 | ,921 | ,034 |
|  | N | 12 | 12 | 12 | 12 | 12 | 12 | 12 |
| I.Mutant | Pearson correlation | -,520 | ,191 | -,207 | ,218 | 1 | ,435 | -,141 |
|  | Sig. (bilateral) | ,083 | ,552 | ,518 | ,495 |  | ,157 | ,662 |
|  | N | 12 | 12 | 12 | 12 | 12 | 12 | 12 |
| MUpro | Pearson correlation | ,003 | -,284 | -,350 | -,032 | ,435 | 1 | ,037 |
|  | Sig. (bilateral) | ,992 | ,372 | ,265 | ,921 | ,157 |  | ,910 |
|  | N | 12 | 12 | 12 | 12 | 12 | 12 | 12 |
| ModPred | Pearson correlation | -,205 | -,274 | ,337 | -,613^*^ | -,141 | ,037 | 1 |
|  | Sig. (bilateral) | ,522 | ,389 | ,284 | ,034 | ,662 | ,910 |  |
|  | N | 12 | 12 | 12 | 12 | 12 | 12 | 12 |
| *.the correlation is significant at the 0.05 level (bilateral).  Supplementary figure 1: correlation analysis among predictions of pathogenic nsSNPs in the *ACE2* gene by using various software tools. | | | | | | | | |
